# MTDH mediates trastuzumab resistance in HER2 positive breast cancer by decreasing PTEN expression through an NFκB-dependent pathway

**DOI:** 10.1186/1471-2407-14-869

**Published:** 2014-11-24

**Authors:** Cheng Du, Xiaomin Yi, Wenchao Liu, Tao Han, Zhaozhe Liu, Zhenyu Ding, Zhendong Zheng, Ying Piao, Jianlin Yuan, Yaling Han, Manjiang Xie, Xiaodong Xie

**Affiliations:** Department of Oncology, General Hospital of Shenyang Military Area Command, Shenyang, 110016 P. R. China; Department of Oncology, Xijing Hospital, Fourth Military Medical University, Xi’an, P. R. China; Department of Urology, Xijing Hospital, Fourth Military Medical University, Xi’an, P. R. China; Department of Urology, PLA 105 Hospital, Hefei, P. R. China; Department of Cardiology, General Hospital of Shenyang Military Area Command, Shenyang, 110016 P. R. China; Key Laboratory of Aerospace Medicine, Ministry of Education, Fourth Military Medical University, Xi’an, 710032 P. R. China

**Keywords:** Metadherin (MTDH), Trastuzumab, Drug resistance, Human epidermal growth factor receptor 2 (HER2), Breast cancer, Phosphatase and tensin homologue deleted from chromosome 10 (PTEN), Nuclear factor kappa B (NFκB)

## Abstract

**Background:**

Trastuzumab resistance is almost inevitable in the management of human epidermal growth factor receptor (HER) 2 positive breast cancer, in which phosphatase and tensin homolog deleted from chromosome 10 (PTEN) loss is implicated. Since metadherin (MTDH) promotes malignant phenotype of breast cancer, we sought to define whether MTDH promotes trastuzumab resistance by decreasing PTEN expression through an NFκB-dependent pathway.

**Methods:**

The correlations between MTDH and PTEN expressions were analyzed both in HER2 positive breast cancer tissues and trastuzumab resistant SK-BR-3 (SK-BR-3/R) cells. Gene manipulations of MTDH and PTEN levels by knockdown or overexpression were utilized to elucidate molecular mechanisms of MTDH and PTEN implication in trastuzumab resistance. For in vivo studies, SK-BR-3 and SK-BR-3/R cells and modified derivatives were inoculated into nude mice alone or under trastuzumab exposure. Tumor volumes, histological examinations as well as Ki67 and PTEN expressions were revealed.

**Results:**

Elevated MTDH expression indicated poor clinical benefit, shortened progression free survival time, and was negatively correlated with PTEN level both in HER2 positive breast cancer patients and SK-BR-3/R cells. MTDH knockdown restored PTEN expression and trastuzumab sensitivity in SK-BR-3/R cells, while MTDH overexpression prevented SK-BR-3 cell death under trastuzumab exposure, probably through IκBα inhibition and nuclear translocation of p65 which subsequently decreased PTEN expression. Synergized effect of PTEN regulation were observed upon MTDH and p65 co-transfection. Forced PTEN expression in SK-BR-3/R cells restored trastuzumab sensitivity. Furthermore, decreased tumor volume and Ki67 level as well as increased PTEN expression were observed after MTDH knockdown in subcutaneous breast cancer xenografts from SK-BR-3/R cells, while the opposite effect were found in grafts from MTDH overexpressing SK-BR-3 cells.

**Conclusions:**

MTDH overexpression confers trastuzumab resistance in HER2 positive breast cancer. MTDH mediates trastuzumab resistance, at least in part, by PTEN inhibition through an NFκB-dependent pathway, which may be utilized as a promising therapeutic target for HER2 positive breast cancer.

**Electronic supplementary material:**

The online version of this article (doi:10.1186/1471-2407-14-869) contains supplementary material, which is available to authorized users.

## Background

The human epidermal growth factor receptor (HER) 2 oncogene from the epidermal growth factor receptor (EGFR) family encodes a receptor tyrosine protein kinase (RTK) that involves in crucial adaptations of cell function under pathophysiological processes [1]. However, HER2 overexpression in cancer cells promotes a malignant phenotype, presented as increased proliferation and invasion, reduced apoptosis, accelerated angiogenesis and enhanced resistance to anticancer therapy [2, 3]. In approximately 20% of invasive breast cancer patients, HER2 overexpression occurs and correlates with shortened disease free survival and overall survival [4, 5].

Trastuzumab, a humanized antibody targeting the extracellular domain of HER2, has been approved for the treatment of HER2-overexpressing breast cancer in both the metastatic and adjuvant settings [6]. Currently, combination therapy using trastuzumab and conventional chemotherapeutic agents were recommended as first line therapy for the management of HER2 positive breast cancer, which significantly improves patient outcomes [7, 8]. However, about 15% of patients with early-stage HER2 positive breast cancer progress to metastatic disease. Besides, most patients who achieve an initial response will develop refractory trastuzumab resistance within one year [9]. Despite several mechanisms of trastuzumab resistance have been proposed, including loss of PTEN activity and up-regulation of the PI3K/Akt pathway, accumulation of a truncated form of the HER2 receptor (p95-HER2), failure to elicit an appropriate immune response and increased signaling from alternative pathways such as EGFR and IGF-1R [10, 11], the detailed mechanism implicated in trastuzumab resistance remains unclear.

Metadherin (MTDH), also named as astrocyte elevated gene-1 (AEG-1) and lysine-rich CEACAM1 coisolated (LYRIC), is a 64 kDa single trans-membrane protein originally cloned as a human immunodeficiency virus (HIV)-1-inducible transcript in primary human fetal astrocytes [12]. MTDH is intensively expressed in many types of cancer, including hepatocellular carcinoma (HCC), breast, prostate, gastric, renal and colorectal cancer, non-small cell lung cancer, esophageal squamous cell carcinoma and glioma, actively participating in cancer invasion, angiogenesis, autophagy and metastasis formation [13–17]. Down-regulation of MTDH reduces cell proliferation and increases apoptosis [18], while MTDH overexpression indicates poor prognosis in invasive breast cancer [19, 20]. Besides, MTDH promotes both chemo- and tamoxifen-resistance [21–25]. However, whether MTDH mediates trastuzumab resistance has not been investigated.

PTEN (phosphatase and tensin homologue deleted from chromosome 10) dephosphorylates the 3’-sites of the phosphoinositides PIP2 and PIP3 that involve in the activation of PI3K/Akt pathway, playing an important role in cellular survival. PTEN expression can be suppressed by tumor necrosis factor-(TNF) through activating NFκB pathway. NFκB comprises a transactivation part RelA/p65 and a DNA-binding part p50 (NFκB1) and p52 that combine with inhibitor of NFκB (IκB) and resides in the cytoplasm and remains inactive in unstimulated circumstances. Upon stimulation, IκB phosphorylation release NFκB, which translocates into the nucleus and activates target genes against apoptosis and death.

In the present study, we tested the hypothesis that MTDH mediates trastuzumab resistance by decreasing PTEN expression through NFκB dependent pathway.

## Methods

### Patients and tissue samples

This study was approved by the Ethics Committee of General Hospital of Shenyang Military Area Command. HER2 positive tissue specimens confirmed by immunohistochemistry were collected with informed consent from 118 female breast cancer patients treated in aforementioned hospital between 2006 and 2011. All the patients were recommended to use trastuzumab-based therapy and 36 patients with advanced disease received trastuzumab-based regimen for the first-line therapy (TCH regimen: Taxotere 75 mg/m^2^, Carboplatin AUC 6 mg/ml/min, and Herceptin 8 mg/kg as initial dose reduced to 6 mg/kg every 3 weeks). Clinical and pathological classification and staging were evaluated according to the American Joint Committee on Cancer criteria. Clinical benefit rate from trastuzumab, defined as patients having a complete response, partial response, or stable disease ≥ 6 month, was evaluated by the Response Evaluation Criteria in Solid Tumors (RECIST, version 1.1). Progression-free survival (PFS) were calculated from the onset of treatment to disease progression or death. Tissues were embedded for immunohistochemical analysis of MTDH and PTEN expression.

### Histological examination

Hematoxylin and eosin (HE) staining for histological analysis was implemented according to previously reported protocol [26]. Briefly, 4-μm sections were stained with hematoxylin and eosin and then observed under Eclipse 80i microscope (Nikon Corp., Japan).

Immunohistochemical analysis was carried out pursuant to the manuals of streptavidin-peroxidase-biotin reagent Kit (ZSBio, Beijing, China). Briefly, 4-μm sections were rehydrated and incubated with anti-MTDH and anti-PTEN antibodies (Cell Signaling Technology, MA, USA) or PBS at 4°C overnight, followed by sequential incubation with biotinylated secondary antibody, streptavidin-horseradish peroxidase complex and diaminobenzidine (DAB). Then slides were counterstained with hematoxylin, dehydrated, and mounted. Sections were observed and imaged under light microscope.

The levels of MTDH and PTEN expression were evaluated based on the staining intensity (SI) and percentage of positively stained tumor cells (PP). SI was defined as: 0 (no staining); 1 (weak staining); 2 (moderate staining) and 3 (strong staining). PP was graded according to the following criteria: 0 (no positive tumor cells); 1 (1%-10% positive tumor cells); 2 (11%-50% positive tumor cells); 3 (51%-70% positive tumor cells); and 4(>70% positive tumor cells). The immunoreactive score (IRS) was calculated as follows: IRS = SI × PP. Low expression was defined as an IRS of 3 or less.

### Cell culture and development of trastuzumab resistance

Human breast cancer cell line SK-BR-3 was obtained from the American Type Culture Collection (ATCC, Manassas, VA, USA). Culture medium was RPMI 1640 containing 10% fetal bovine serum (Gibco, NY, USA), supplemented with 100U/mL penicillin and 100 μg/mL streptomycin (Sigma-Aldrich, MO, USA). Cells were cultured in humidified atmosphere containing 5% CO_2_ at 37°C. Resistant cells (SK-BR-3/R) were developed by culturing parental SK-BR-3 cells in the presence of 5 μg/ml trastuzumab (Genentech, CA, USA) for 8 months. Trastuzumab was dissolved in sterile apyrogen water and stored at 4°C before use.

### RNA extraction and real-time RT-PCR

Total RNA was isolated using TRIzol reagent (Life Technologies, CA, USA) and reversely transcribed with a reverse transcription polymerase chain reaction (PCR) kit (Takara, Dalian, China) as described by the manufacturers. Real-time PCR was done using specific primers (MTDH: 5’-AAATAGCCAGCCTATCAAGACTC-3’ and 5’-TTCAGACTTGGTCTGTGAAGGAG-3’; PTEN: 5’- AATCCTCAGTTTGTGGTCT-3’ and 5’-GGTAACGGCTGAGGGAACT-3’; GAPDH: 5’-GACTCATGACCACAGTCCATGC-3’ and 5’-AGAGGCAGGGATGATGTTCTG-3’) with the QuantiTect SYBR Green PCR Kit (Takara, Dalian, China) as described elsewhere [27].

### Western blot analysis

Proteins were extracted using NE-PER® Nuclear and Cytoplasmic Extraction Reagents (Pierce, Rockford, IL, USA) containing protease inhibitors and phosphatase inhibitors. Proteins were quantified using the BCA protein assay kit (Thermo, IL, USA) and separated using NuPAGE 4-12% Bis-Tris gel (Invitrogen, CA, US). After transferred to PVDF membrane (Millipore, MA, USA), proteins were detected by the following antibodies: mouse anti MTDH mAb, mouse anti PTEN mAb, rabbit anti AKT mAb, rabbit anti p-AKT(Ser473) mAb (Cell Signaling Technology, MA, USA), β-actin antibody (Abcam, UK) and anti-mouse or anti-rabbit secondary antibody (Abcam, UK). Blots were finally visualized using an enhanced chemiluminescence detection kit (Thermo, IL, USA).

### Immunofluorescence staining

Cells were seeded on glass cover slips and fixed with 4% formaldehyde in PBS. Immunofluorescence staining was done with anti-MTDH, anti-PTEN, anti-p-AKT and anti-p65 (Cell Signaling Technology, MA, USA) antibodies. Slips were incubated with indicated primary antibodies, followed by incubation with fluorescein isothiocyanate-conjugated goat anti-mouse secondary antibody or Texas Red-conjugated goat-anti-rabbit secondary antibody (Abcam, Cambridge, MA, USA). Then slips were counterstained with 4’,6-diamidino-2- phenylindole dihydrochloride (DAPI) solution (Sigma, St Louis, MO, USA). Slips were observed and imaged under fluorescence microscope.

### Cell proliferation assay

Cell proliferation was measured by methylthiazolyltetrazolium (MTT) assay and 5-ethynyl-2′-deoxyuridine (EdU) incorporation assay. For MTT assay, cells were seeded at a density of 3000 cells per well in 96-well plates and regrew for 24 h. Varied concentrations of trastuzumab (0, 0.63, 1.25, 2.5, 5, 10, 20 μg/ml) or equal volumes of sterile apyrogen water were added and cocultured for 1 to 7 days. Cells were incubated subsequently with 5 mg/ml MTT (Sigma) and 150ul of dimethylsulfoxide (DMSO). The absorbance was measured at 570 nm. For EdU incorporation assay, staining procedure was performed using Cell-Light™ EdU Apollo® 488 In Vitro Imaging Kit (RBbio, Guangzhou, China). Briefly, cells in 96-well plates were exposed to 5 μg/ml trastuzumab for 4 days. After incubation with EdU working solution for 2 h, cells were stained with DAPI solution. EdU-labeled cells was counted in ten randomly selected fields under fluorescent microscope Olympus BX51. Cell proliferation rate was calculated as a percentage to the control group.

### TUNEL assay

To detect cell apoptosis, terminal deoxynucleotidyl transferase-mediated dUTP nick end labeling (TUNEL) assay was performed using the In Situ Cell Death Detection Kit according to the instruction manual of the manufacturer (Roche, Mannheim, Germany). Briefly, samples were fixed using 4% paraformaldehyde. After membrane penetration with 0.1% Triton X-100 and PBS wash, samples were incubated with TUNEL reaction mixture for 60 min at 37°C. DAPI was utilized for nuclei counterstain. Then samples were observed and imaged under fluorescence microscope. Cells transfected GFP-PTEN were treated with POD substrates and diaminobenzidine (DAB) after incubation with TUNEL reaction mixture. Then cells were counterstained with hematoxylin and imaged under light microscope. Apoptosis rate was calculated as a percentage to the control group.

### Retrovirus infection and plasmid transfection

MTDH knockdown was achieved with a pGV112-MTDH-shRNA system (Genechem Co. Ltd. Shanghai, China) targeting the following sequence: 5’-CAGAAGAAGAAGAACCGGA-3’ as reported by Yoo’s group [28]. Vectors expressing a non-targeting scrambled shRNA were used as control. MTDH overexpression was achieved using the retroviral expression vector pReceiver-Lv105 (GeneCopoeia Rockville, MD, USA). Viruses were generated and used to infect target cells as previously described [29]. The stably infected cells were selected with 0.5 μg/ml puromycin. Western blot analysis was performed to validate the knockdown or overexpression of MTDH.

The p-CMV/Neo-PTEN and p-CMV/Neo-p65 expression constructs were purchased from GeneCopoeia (Rockville, MD, USA). The pU6/Neo-p65 shRNA (p65 shRNA) that targets the sequence GCCCTATCCCTTTACGTCA and the shRNA scrambled control clone for pU6/Neo were obtained from GenePharm Co. Ltd. (Shanghai, China). Transfection was performed using Lipofectamine 2000 (Qiagen, KJ Venlo, NL, USA) according to the manufacturer’s instructions.

### Luciferase reporter assay

Before luciferase reporter assay, SK-BR-3 and SK-BR-3/R cells were infected with previously described viruses or their corresponding vectors for 72 hours. The human PTEN promoter was cloned into the pGL4 luciferase reporter vector (PTEN-Luc, Promega, CA, USA). Infected SK-BR-3 (SK-BR-3/R) cells were cotransfected with p-CMV/Neo-p65 (psi-U6/Puro-p65 shRNA) and PTEN-Luc, together with a pGL4 vector, which expresses renilla luciferase as an internal transfection control for transfection efficiency. The expression of firefly and renilla luciferases was analyzed 48 h after transfection using the Dual-Luciferase®Reporter (DLR™) Assay System (Promega, CA, USA) according to the manufacturer’s instructions. Relative luciferase activity was expressed as the firefly luciferase activity normalized with respect to the renilla luciferase activity.

### In vivo experiments

Animal experiments were approved by the Laboratory Animal Ethics Committee of the Fourth Military Medical University and were conducted in accordance with the Animal Research: Reporting *In Vivo* Experiments (ARRIVE) guidelines. 48 female athymic nude mice (4–6 weeks old, 18-25 g) were purchased from experimental animal center of the Fourth Military Medical University. MTDH-knockdown SK-BR-3/R cells (1 × 10^7^ cells in 100 μl 50% Matrigel) or MTDH overexpression SK-BR-3 cells were inoculated subcutaneously into the mammary fat pads of mice as previously described [30]. SK-BR-3/R cells and SK-BR-3 cells were served as control, respectively. Three weeks later, 12 mice in each group received i.p injection of 100 μl trastuzumab solution (10 mg/kg, n = 6) or 100 μl sterile PBS (n = 6) twice weekly. Tumor xenografts in each group were measured with calipers every week. Tumor volume in mm^3^ was calculated by the formula: volume = width^2^ × length/2 [31]. Mice were sacrificed at week 5. Tumor xenografts were retrieved for histological examination and immunohistochemical analysis of PTEN and Ki67 expressions.

### Statistical analysis

Numerical data were presented as mean ± standard deviation. The correlations between MTDH expression and clinical factors were evaluated by Chi square test or Fisher’s exact test. Associations between variables were analyzed using the Spearman correlation test. PFS were compared using the Kaplan–Meier method with the log-rank test. Comparisons for numerical data were performed using a two-tailed Student’s t test. All statistical analyses were carried out using the SPSS 16.0 statistical software (SPSS Inc., Chicago, IL, USA). P value less than 0.05 was considered statistically significant.

## Results

### MTDH overexpression induced trastuzumab resistance in HER2 positive breast cancer patients

**Figure 1:**
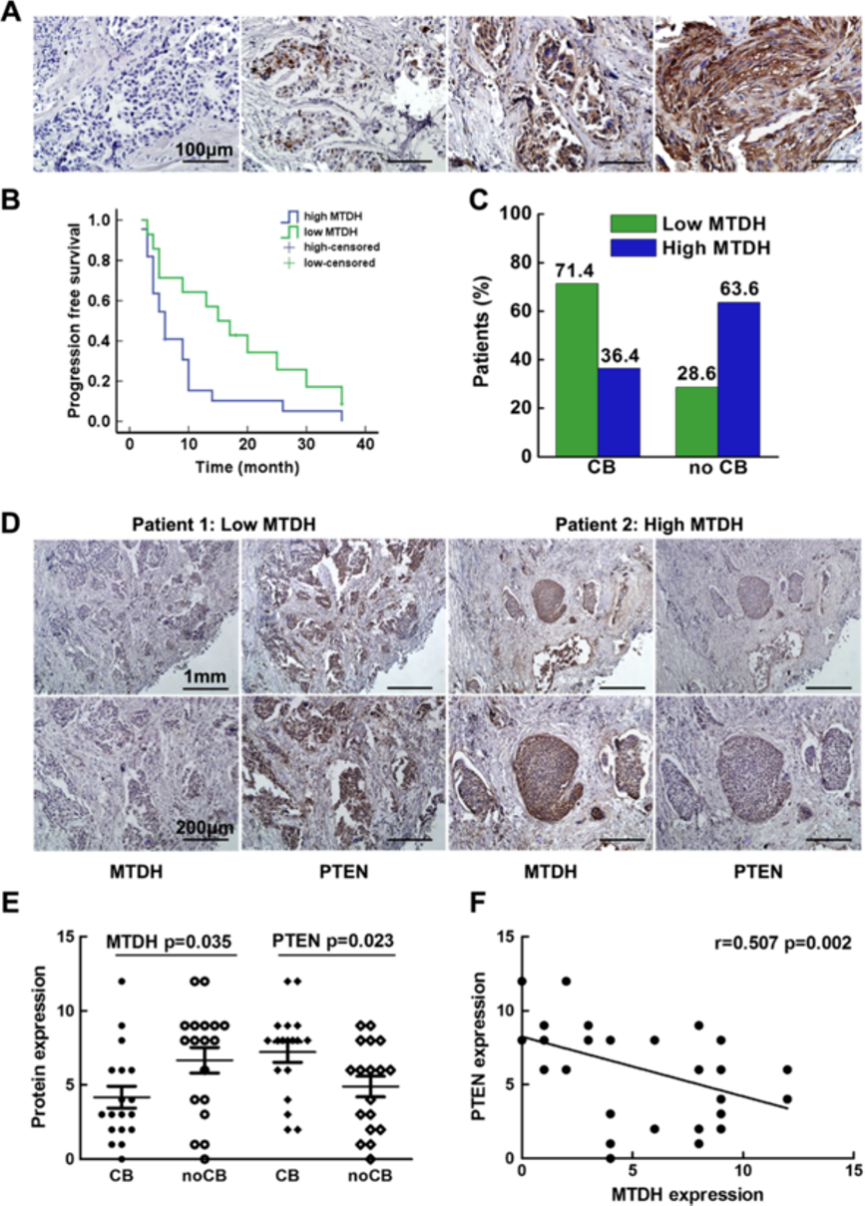
**MTDH expression in HER2 positive breast cancer patients was associated with PTEN reduction and trastuzumab resistance. A**. Representative image of MTDH immunohistochemical staining in paraffin-embedded tissue from118 breast cancer patients (IRS =0, 3, 6, 12, magnification at 400×, scale bar =100 μm); **B**. The comparison of PFS between patients with high MTDH expression or low MTDH expression. Median PFS was 6 months in patients with high MTDH expression and 15 months with low MTDH expression. Log Rank test P = 0.024; Patients were treated with trastuzumab-based first line therapy; **C**. Different clinical benefit (CB) rates in two subgroups, high MTDH expression (n =22) and low MTDH expression (n =14) were found (P =0.025); **D**. MTDH level was negatively correlated with PTEN expression in patients treated with trastuzumab-based first line therapy; **E**. The scatter plot of MTDH and PTEN expressions in patients with different clinical benefits; **F**. The regression analysis of MTDH and PTEN expressions.

Detailed characteristics of the 118 patients with HER2 positive breast cancer were summarized in Table 1. High MTDH expression was found in over half of these patients (62.7%). There were varied MTDH expressions in patient subgroups classified by positive nodal status (P = 0.026), advanced pathological stage (P = 0.012) and high Ki67 index (P = 0.033); however, there was no association between MTDH expression and age, hormone receptor status, or histological grade. As revealed in Figure 1A, MTDH expression in tumors was heterogeneous and MTDH had both cytoplasmic and nuclear localizations. Subgroup analyses were further performed to investigate the MTDH expression in 36 patients who received trastuzumab-based first line therapy. Clinical benefit from trastuzumab was defined as patients having a complete response, partial response, or stable disease ≥ 6 months. High MTDH expression was detected in 22 patients, of whom only 8 patients (36.4%) acquired clinical benefit and the median PFS was 6 months. In contrast, 10 in 14 patients (71.4%) with low MTDH expression gained clinical benefit and a median PFS 15 months (Figure 1B and C). There was a trend toward a higher clinical beneficial rate and a longer PFS in patients with low MTDH expression (P = 0.024).

We further examined the association of MTDH and PTEN expression in these 36 patients who received trastuzumab-based therapy. They were divided into two subgroups according to the treatment response. Interestingly, MTDH expression was significantly lower in patients who achieved clinical benefit from trastuzumab-based therapy than those with progressive disease (P = 0.035). In contrast to decreased MTDH level, PTEN expression was significantly higher (P = 0.023) in these patients as compared with patients with progressive diseased (Figure 1E). Moreover, High MTDH level was correlated with low PTEN expression in patients with poor clinical benefit, confirmed by regression analysis (Figure 1F, r = 0.507, P = 0.002). These results suggest that MTDH up-regulation is associated with PTEN reduction and trastuzumab resistance in HER2 positive breast cancers.

**Table 1:** **Patient characteristics**

Variable	Total no.	MTDH expression				*P*value*
		High		Low		
		No.	%	No.	%	
Age						
<50	40	24	20.3	16	13.6	
≥50	78	50	42.4	28	23.7	0.663
Nodal status						
Positive	82	58	49.2	24	20.3	
Negative	36	16	13.6	20	16.9	0.007
Stage						
I	20	7	5.9	13	11.0	
II	27	17	14.4	10	8.5	
III	25	18	15.3	7	5.9	
IV	46	32	27.1	14	11.9	0.038
Grade						
1	28	14	11.9	14	11.9	
2	43	31	26.3	12	10.2	
3	47	29	24.5	18	15.2	0.167
ER status						
Positive	66	40	33.9	26	22.0	
Negative	52	34	28.8	18	15.3	0.594
PR status						
Positive	60	36	30.5	24	20.3	
Negative	58	38	32.2	20	17.0	0.536
Ki67						
High index	78	52	44.1	23	19.5	
Low index	40	22	18.6	21	17.8	0.015

*Chi square test or Fisher’s exact test.

### MTDH overexpression and PTEN reduction protected trastuzumab-resistant HER2 positive breast cancer cells from trastuzumab exposure

**Figure 2:**
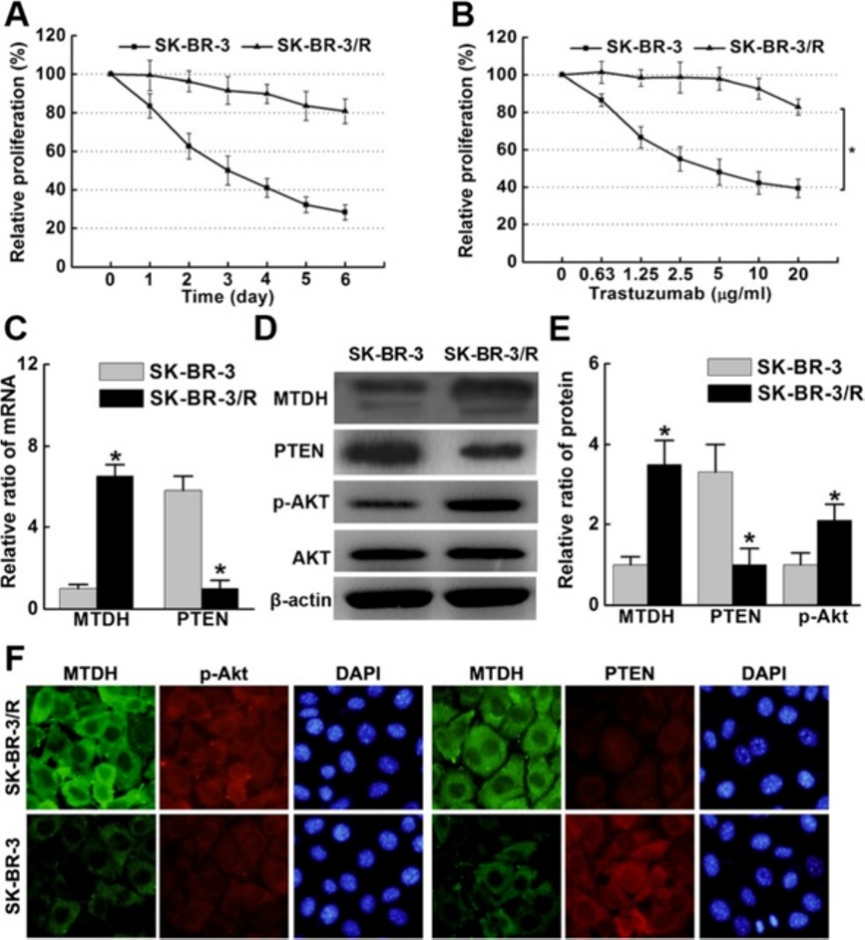
**Elevated MTDH and reduced PTEN expressions in trastuzumab-resistant HER2 positive breast cancer cells. A**. Relative proliferation of SK-BR-3 and SK-BR-3/R cells under trastuzumab exposure (5 μg/ml) at different time points by MTT assay (*P < 0.05); **B**. Dose-dependent trastuzumab cytotoxicity in SK-BR-3 cells by MTT assay (*P < 0.05). The proliferation curve of SK-BR-3/R cells was relatively flat, indicating trastuzumab resistance; **C**. Real-timeRT-PCR revealed mRNA expression of MTDH is significantly higher in SK-BR-3/R cells, while PTEN expression on the opposite (*P < 0.05); **D**. Higher levels of MTDH and p-Akt expressions as well as reduced PTEN expression were detected in SK-BR-3/R cells by western blot analysis; **E**. Significant differences in MTDH, PTEN and p-AKt expressions between SK-BR-3 and SK-BR-3/R cells (*P < 0.05); **F**. The immunofluorescence staining of SK-BR-3 and SK-BR-3/R cells revealed differential MTDH, PTEN and p-Akt expressions. All in vitro experiments were repeated for three times.

After continuous exposure to 5 μg/ml trastuzumab for 8 months, HER2 overexpressing breast cancer cells with trastuzumab resistant (SK-BR-3/R) was successfully developed. Relative proliferation in SK-BR-3/R cells under trastuzumab exposure at different time points was higher than in SK-BR-3 cells. Dose-dependent trastuzumab cytotoxicity in SK-BR-3 cells was observed, but not obvious in SK-BR-3/R cells (Figure 2A and B). The relative ratio of MTDH mRNA was significantly higher in SK-BR-3/R cells. In contrast, PTEN mRNA was significantly lower in SK-BR-3/R cells compared with their parental counterparts (Figure 2C). Moreover, elevated MTDH and p-Akt, but reduced PTEN expressions were found in SK-BR-3/R cells than in parental cells by western blot analysis (Figure 2D and E). These findings were further supported by immunofluorescence observance (Figure 2F), suggesting the implication of MTDH and PTEN/Akt signaling in trastuzumab resistance.

### Alterations of MTDH expression regulated trastuzumab resistance via modulating PTEN/Akt signaling in HER2 positive breast cancer cells

**Figure 3:**
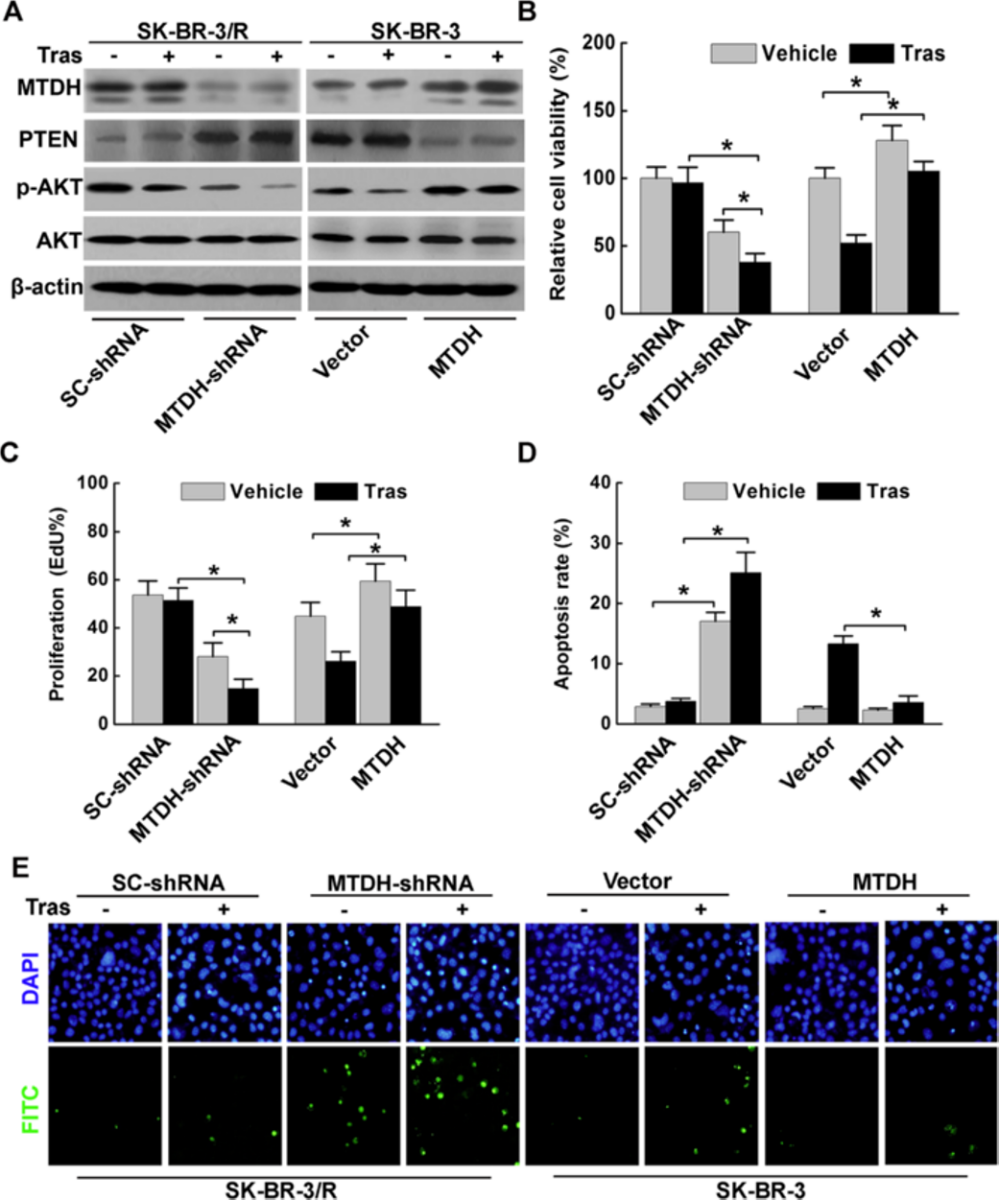
**MTDH manipulation regulated trastuzumab resistance by influencing PTEN and pAkt expression in HER2 positive breast cancer cells. A**. Western blot analysis revealed that MTDH-shRNA blocked MTDH expression accompanied by increased PTEN and reduced p-Akt in SK-BR-3/R cells. Up-regulation of MTDH in SK-BR-3 cells was also realized accompanied with downregulated PTEN and elevated p-Akt; **B**. MTT assay indicated that MTDH-shRNA significantly reduced trastuzumab resistance in SK-BR-3/R cells, while MTDH up-regulation led to the resistance of trastuzumab (5 μg/ml, 72 h) in SK-BR-3 cells (*P < 0.05). MTDH was overexpressed in SK-BR-3 cells and knocked down in SK-BR-3/R cells; **C**. 5-ethynyl-2′-deoxyuridine (EdU) incorporation assay demonstrated trastuzumab resistance in MTDH-SK-BR-3 cells but not in engineered SK-BR-3/R cells (*P < 0.05); **D**. and **E**. Results of TUNEL assay denoted higher apoptosis rate in SK-BR-3/R cells interfered by MTDH-shRNA (magnification at 200×, *P < 0.05).

To investigate whether MTDH manipulation modulate trastuzumab sensitivity, MTDH-shRNA was delivered into SK-BR-3/R cells and MTDH into SK-BR-3 cells, respectively. As shown in Figure 3A, MTDH-shRNA effectively blocked MTDH expression in SK-BR-3/R cells in comparison with control group that were infected with scrambled shRNA. Moreover, MTDH-shRNA increased PTEN expression and reduced Akt phosphorylation, suggesting the inactivation of Akt signaling. Next, we examined the impact of MTDH-shRNA on SK-BR-3/R cell survival under trastuzumab exposure. Down-regulation of MTDH decreased the viability (Figure 3B) and proliferation (Figure 3C and Additional file 1: Figure S1) of SK-BR-3/R cells. In addition, significant increase of apoptosis rate was observed (Figure 3D). Importantly, trastuzumab sensitivity was recovered in SK-BR-3/R cells infected with MTDH-shRNA since trastuzumab resumed efficiency at the concentration of 5 μg/ml, as demonstrated by the results of cell proliferation and TUNEL assay.

We sought to define whether MTDH overexpression in SK-BR-3 cells contribute to trastuzumab resistance using a retrovirus MTDH delivery system. In contrast to MTDH suppression in trastuzumab resistant cells, overexpressing MTDH in parental SK-BR-3 cells decreased PTEN expression and increased Akt phosphorylation (Figure 3A). Increased MTDH expression was detected in MTDH-SK-BR-3 cells compared to SK-BR-3 cells infected with empty vectors (Figure 3A). In addition, overexpressed MTDH not only enhanced the viability (Figure 3B and Additional file 1: Figure S1) and proliferation (Figure 3C) in MTDH-SK-BR-3 cells, but also reduced the cell apoptosis (Figure 3D). Surprisingly, MTDH-SK-BR-3 cells acquired resistance to trastuzumab at the concentration of 5 μg/ml. These evidence supported that MTDH participated in the alteration of trastuzumab resistance via modulating PTEN expression in HER2 positive breast cancer cells.

### MTDH modulated PTEN expression via NFκB signaling pathway

PTEN is tightly controlled by various non-genomic mechanisms, such as transcriptional regulation and post‑transcriptional regulation by non‑coding RNAs besides PTEN mutation or deletion. Increasing evidence demonstrates that NFκB signaling directly or indirectly inhibits PTEN transcription. It is reported that MTDH activate NFκB by promoting degradation of IκBα and nuclear translocation of p65. In these contexts, we postulated that MTDH modulate PTEN expression via NFκB signaling pathway.

**Figure 4:**
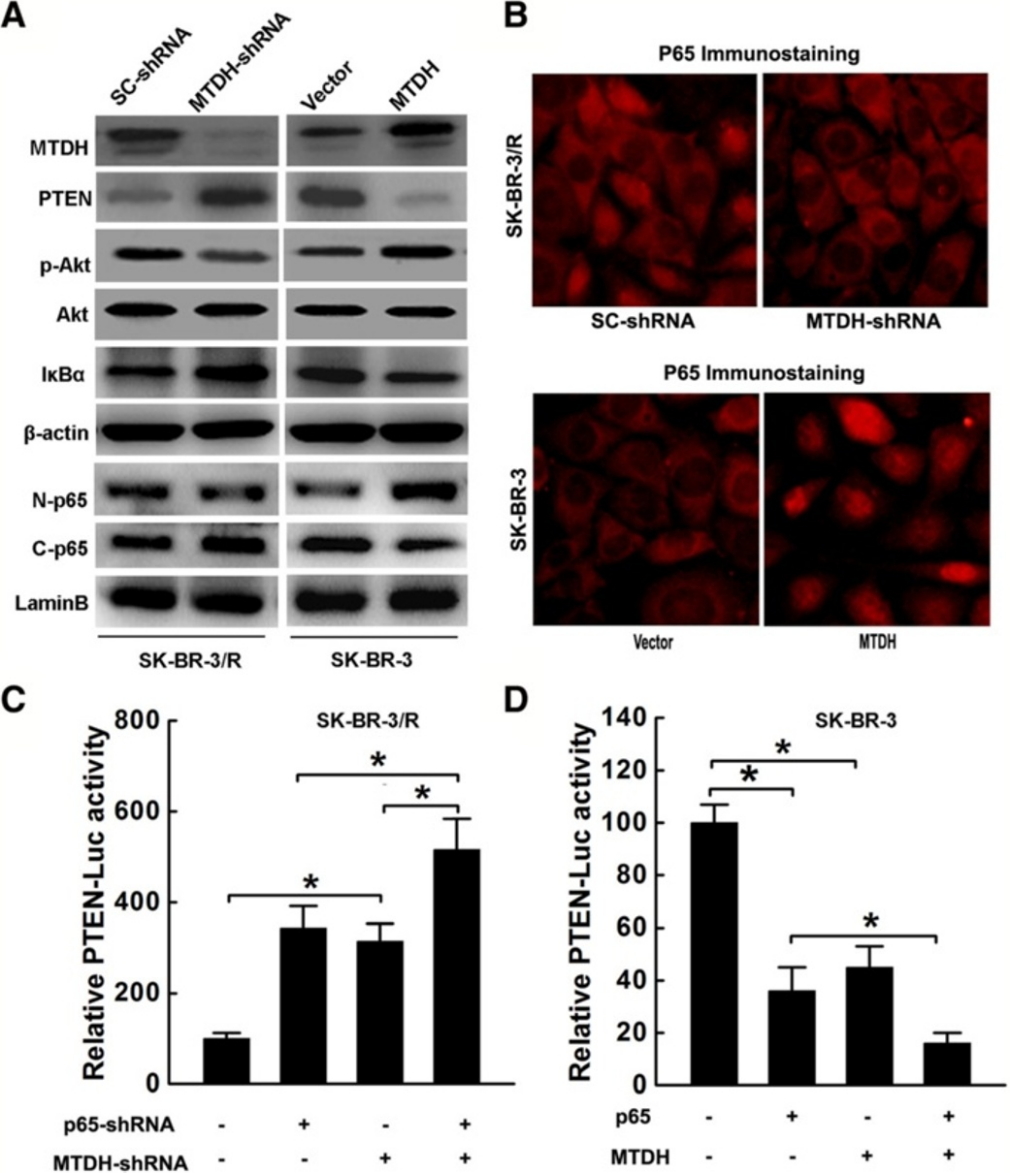
**MTDH modulated PTEN expression via NF-κB signaling pathway. A**. MTDH downregulation resulted in increased IκBαexpression and decreased p65 nuclear translocation in SK-BR-3/R cells. High level of PTEN and decreased p-Akt were also observed. MTDH overexpression activated NF-κB by promoting IκBα degradation and p65 nuclear translocation in SK-BR-3 cells. Decreased PTEN expression and high level of p-Akt were observed. β-actin and lamin **B**. were subcellular loading controls for cytoplasmic and nuclear extracts respectively; **B**. Immunofluorescence imagings of p65 in cells with MTDH manipulation (magnification at 400×); **C**. Relative PTEN-Luc activity after co-transfection of p65 and MTDH-shRNA in SK-BR-3/R cells (*P < 0.05); **D**. Relative PTEN-Luc activity after co-transfection of p65 and MTDH in SK-BR-3 (*P < 0.05).

Based on this hypothesis, we evaluated the expressions of IκBα and p65 by western blot analysis in both MTDH-SK-BR-3 cells and MTDH-shRNA infected SK-BR-3/R cells. As shown in Figure 4A, the level of IκBα increased significantly 72 hours after MTDH-shRNA infection in SK-BR-3/R cell compared with scrambled-shRNA infected cells. Meanwhile, the levels of p65 increased in cytoplasmic extract and decreased in the nuclear extract of cells after MTDH-shRNA infection. In contrast, the level of IκBα increased significantly after MTDH infection, compared with empty vector infected SK-BR-3 cells. Consistently, the levels of p65 protein decreased in cytoplasmic extract and increased in the nuclear extract of cells after MTDH infection. Immunofluorescence staining confirmed the nuclear translocation of p65 following MTDH and MTDH-shRNA infection (Figure 4B).

Next, we performed transient co-transfection experiments to examine whether MTDH regulates PTEN promoter activity via inducing p65 translocation in SK-BR-3 and SK-BR-3/R cells. SK-BR-3 cells were either infected with MTDH or vector and then transfected with p65 and PTEN-Luc containing NFκB binding sites upstream of the luciferase gene. Both MTDH infection and p65 transfection suppressed PTEN-Luc activity in SK-BR-3 cells (Figure 4C). Furthermore, PTEN-Luc suppression by p65 was enhanced by infection of MTDH. On the other hand, SK-BR-3/R cells were either infected with MTDH-shRNA or scrambled shRNA and then transfected with p50-shRNA and PTEN-Luc. MTDH-shRNA infection or p65-shRNA transfection increased PTEN-Luc activity in SK-BR-3/R cells. Additionally, PTEN-Luc induction by p65-shRNA was enhanced by infection of MTDH-shRNA (Figure 4D). All these suggests that p65 suppresses PTEN promoter activity, and MTDH enhances PTEN transcriptional suppression by promoting p65 translocation.

### Forced PTEN expression in SK-BR-3/R cells restored trastuzumab sensitivity

**Figure 5:**
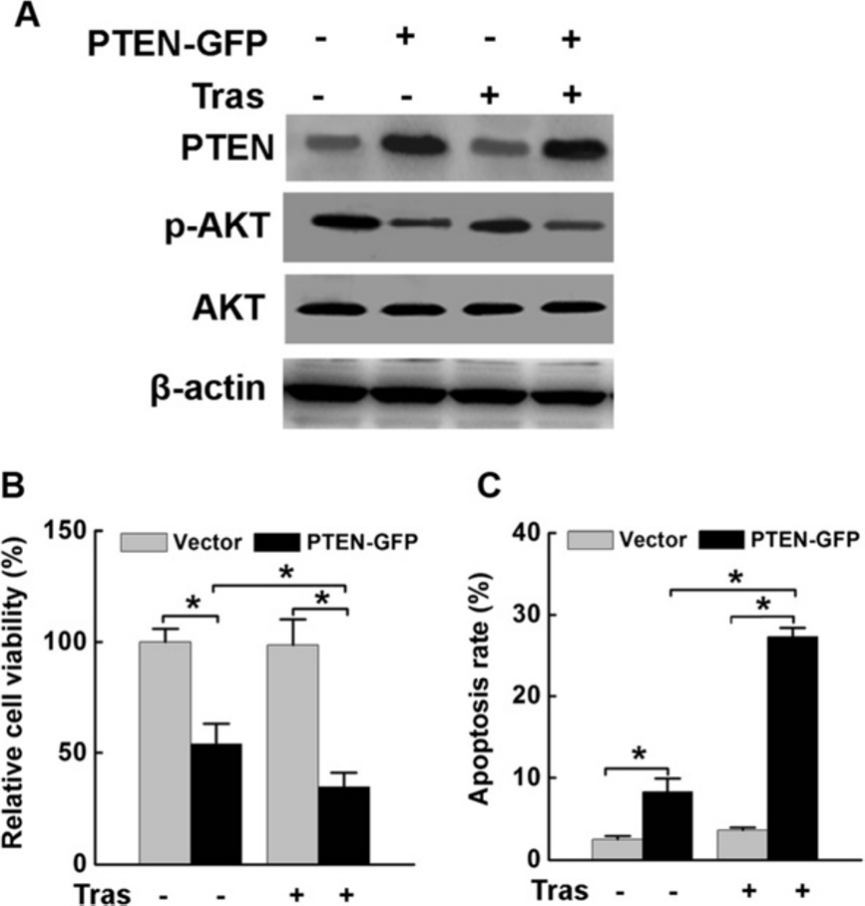
**Forced PTEN expression in SK-BR-3/R cells restored trastuzumab sensitivity. A**. PTEN expression were retrived and p-Akt was decreased in SK-BR-3/R cells transfected with GFP-PTEN; **B**. MTT assay indicated that ectopic PTEN expression in SK-BR-3/R cells reduced cell viability in the absence of trastuzumab. When trastuzumab was added, cell viability was further inhibited (*P < 0.05); **C**. TUNEL assay suggested that ectopic expression of PTEN resensitized the trastuzumab-treated SK-BR-3/R cells to apoptosis (*P < 0.05).

To assess whether restoring PTEN expression in SK-BR-3/R cells may re-sensitize the cancer cells to trastuzumab treatment. We transfected SK-BR-3/R cells with plasmid vector encoding GFP-PTEN and determined the expression of ectopic GFP-PTEN expression after 48 h of transfection by Western blot analysis. Ectopic GFP-PTEN expression driven by a heterologous promoter was not affected by elevated MTDH in SK-BR-3/R cells. (Figure 5A). Aligned with increased PTEN expression, Akt phosphorylation was reduced in these cells. Furthermore, SK-BR-3/R cells with GFP-PTEN transfection restored trastuzumab sensitivity, since the cell viability decreased by 65.3% (Figure 5B) and the cell apoptosis rate increased by 6.5 fold as compared with cells transfected with an empty vector (Figure 5C). Together, these data suggested restoring PTEN expression re-sensitize SK-BR-3/R cells to trastuzumab treatment.

### MTDH contributed to trastuzumab resistance in breast cancer mice model by decreasing PTEN expression

**Figure 6:**
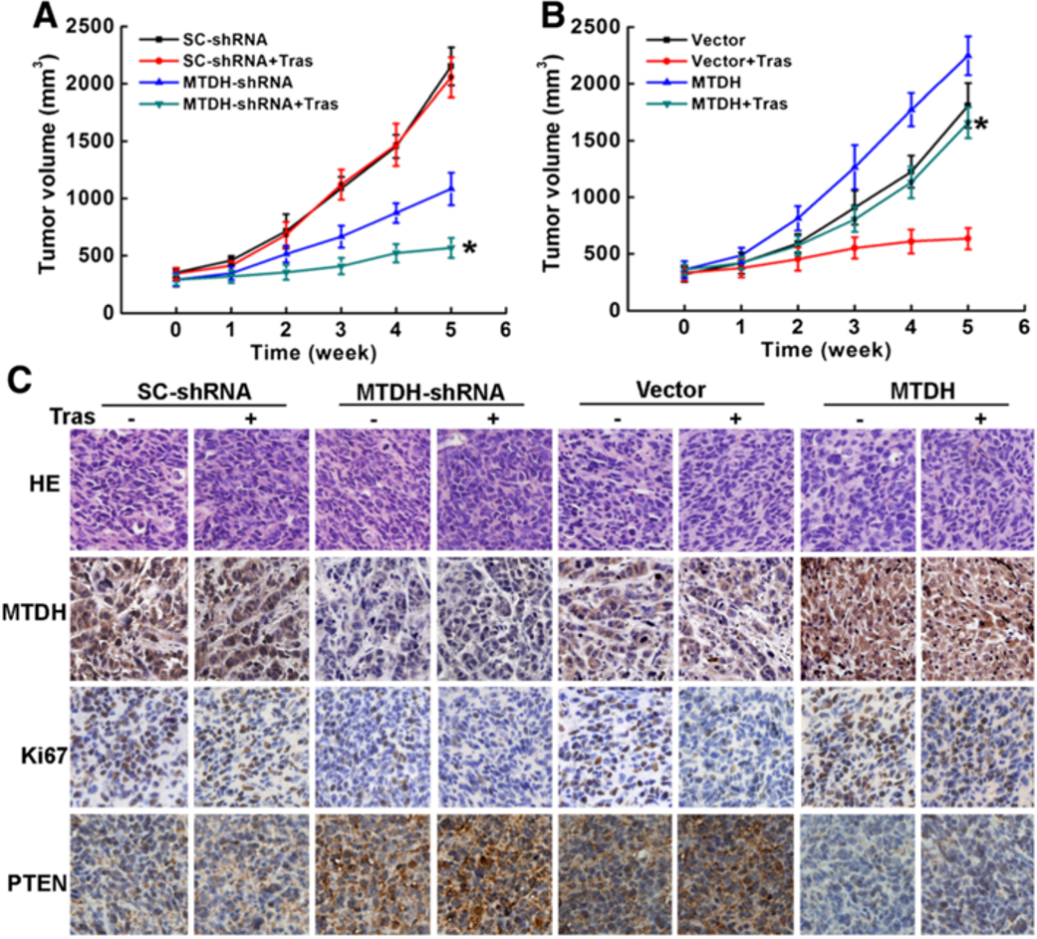
**MTDH contributed to trastuzumab resistance by decreasing PTEN expression in breast cancer model using athymic nude mice. A**. Smaller tumor volumes in MTDH-shRNA group with trastuzumab injection in comparison with vector control group using SK-BR-3/R cells infected with scrambled shRNA (*P < 0.05); **B**. Tumor xenografts in MTDH-SK-BR-3 group outgrew other 3 groups. Compared with vector control group, tumor volumes were larger in MTDH-SK-BR-3 group than that in Vector-SK-BR-3 group treated with trastuzumab (*P < 0.05); **C**. Histological examination of tumor xenografts. Hematoxylin and eosin (HE) staining confirmed the existence of tumor and immunohistochemical analysis revealed distinct Ki67 and PTEN expressions in xenografts (magnification at 400×).

Athymic nude mice bearing breast cancer xenografts were used to demonstrate whether MTDH-mediated PTEN silence still exerted a pivotal role in trastuzumab resistance *in vivo*. Three weeks after subcutaneous inoculation of breast cancer cells, smaller tumor volumes in MTDH-shRNA group with trastuzumab injection in comparison with vector control group using SK-BR-3/R cells infected with scrambled shRNA (Figure 6A and Additional file 2: Figure S2). Tumor xenografts in MTDH-SK-BR-3 group outgrew other 3 groups. Compared with vector control group, tumor volumes were larger in MTDH-SK-BR-3 group treated with trastuzumab (Figure 6B and Additional file 2: Figure S2). Trastuzumab treatment hardly affected tumor growth in SK-BR-3/R group. On the contrary, trastuzumab retarded the growth of tumor xenografts in MTDH-shRNA group and SK-BR-3 group. Histological examination of xenografts retrieved 5 weeks after trastuzumab application confirmed the existence of tumor in these mice. Besides, immunohistochemical analysis revealed diverse PTEN and Ki67 expressions among these groups (Figure 6C and Additional file 2: Figure S2). Reduced PTEN and increased Ki67 expressions were found in MTDH-SK-BR-3 group with or without trastuzumab treatment, while few Ki67 but distinct PTEN stainings were observed in MTDH-shRNA group with trastuzumab injection.

## Discussion

Trastuzumab is a humanized monoclonal antibody targeting the extracellular domain IV of HER2 and is commonly applied for HER2-positive patients. However, trastuzumab resistance is an intractable problem for both clinicians and patients since it denotes a poor prognosis that severely threatens patients’ life. Elucidating the underlying molecular mechanisms implicated in the resistance transition may renew the strategies to fight against refractory HER2 positive breast cancer. A wide range of putative mechanisms to trastuzumab resistance based on laboratory experiments have been proposed but most of them still lack clinical relevance [32, 33].

Results of immunohistochemical studies from several groups reveal diverse MTDH expressions in HER2 positive breast cancer tissues (ranging from 39% to 65%), emphasizing the potential role of MTDH in tumor progression [23, 27, 29, 34]. Moreover, increased MTDH expression correlates with clinicopathological features including positive nodal status, distant metastasis, advanced stage, Ki-67 index and predicts a poor patient survival [27, 34–36]. Besides, MTDH overexpression increases resistance of HCC cells to fluorouracil [28]. It is also recognized that MTDH involves in the resistance of chemotherapeutic drugs in many other types of cancer, probably through various signaling pathways including PI3K/AKT, Wnt/b-catenin, and NFκB [21, 37, 38]. Hu et al. described that knockdown of MTDH decreased the expressions of chemoresistance genes and sensitized cancer cells to a broad spectrum of chemotherapy drugs, including paclitacxel, doxorubicin and cisplatin [29]. In addition, miR-375 that directly inhibited MTDH expression reversed both tamoxifen resistance and accompanying epithelial-mesenchymal transition like properties in tamxifen resistant breast cancer cells [39]. In consistence with previous findings, clinical data in this study revealed high MTDH but low PTEN expressions in HER2 positive breast cancer tissues, and MTDH overexpression was related with advanced pathological stage and Ki-67 index. Moreover, we found that MTDH reversely correlated with PTEN expression. Subgroup analyses proves that MTDH is implicated in trastuzumab resistance in HER2 positive breast cancer, which is further confirmed by elevated MTDH but reduced PTEN expressions in trastuzumab-resistant breast cancer cells. Subsequent experiments demonstrated MTDH mediates trastuzumab resistance by decreasing PTEN expression through an NFκB-dependent pathway, suggesting the potential role of MTDH as a predictive factor of treatment response to anti-HER2 therapy.

The PTEN-PI3K/Akt pathway attracts extensive attentions as a target in refractory HER2 positive breast cancer. Blockade of the PI3K/Akt pathway by PTEN has been reported to induce cell death and apoptosis in trastuzumab resistant breast cancer cells [40–43]. Upon MTDH modulation in SK-BR-3 and trastuzumab-resistant SK-BR-3/R cells, we confirmed that MTDH overexpression first activated NFκB signal and then influenced PTEN expression, thus maintaining PI3K/Akt signaling pathway in HER2 positive breast cancer, which conferred a survival advantage under trastuzumab exposure. On the other hand, MTDH down-regulation suppressed the proliferation potential of SK-BR-3/R cells and restored the sensitivity to trastuzumab, which was in accord with previous findings [9, 10, 36, 44]. Furthermore, forced PTEN expression in SK-BR-3/R cells that was not affected by MTDH level, inhibited PI3K/Akt signaling and restored the trastuzumab sensitivity. All these supported that acquired resistance to trastuzumab in SK-BR-3R cells was regulated, at least in part, by MTDH via influencing PTEN-PI3K/Akt signaling in an NFκB dependent pathway.

Various down-stream molecules of PTEN-PI3K/Akt signaling pathway involving in trastuzumab resistance have also been characterized. Chakrabarty et al. found that trastuzumab-resistant cells relied on a HER2-PI3K-FoxO-Survivin axis [45]. Wu and colleagues reported that blocking the constitutively active Akt significantly increased FOXO1A expression and rendered the cells vulnerable to trastuzumab incubation [46]. In Li’s study, FOXO1 could be downregulated by promoting FOXO1 phosphorylation via the PI3K/Akt pathway, as a consequence, changing the expression pattern of cyclin-dependent kinase inhibitors. Therefore, it is possible to achieve durable responses in breast cancer refractory to trastuzumab therapy by MTDH inhibition, which functions through PTEN-PI3K/Akt pathway to realize tumor regression [40]. Xu and associates reported that MTDH mediated tamoxifen resistance through PTEN-PI3K/Akt pathway in MCF-7 cells [47], but their study lacks the evidence to reveal the interconnection and crosstalk between MTDH and PTEN, which was complemented in our study. Given that PTEN is involved in both tamoxifen-resistance and trastuzumab-resistance [48–50], more studies are needed to validate whether MTDH is a general or a specific mechanism of drug resistance and how it functions in breast cancer.

There are some limitations in our study. Retrospective data were collected from a single center and patient population was not large to form a solid evidence for interpretation and immediate clinical translation.

## Conclusions

MTDH overexpression confers trastuzumab resistance in HER2 positive breast cancer patients, which may also be a potential predictive factor for the evaluation of clinical response to trastuzumab-based anticancer therapy. MTDH mediates trastuzumab resistance, at least in part, through PTEN-PI3K/Akt signaling in an NFκB dependent pathway in HER2 positive breast cancer. Future studies are needed to broaden MDTH inhibition as a therapeutic approach for the treatment of trastuzumab resistant HER2 positive breast cancer.

## Electronic supplementary material

Additional file 1: Figure S1: Representative images of 5-ethynyl-2'-deoxyuridine (EdU) incorporation assay. MTDH was overexpressed in SK-BR-3 cells and knocked down in SK-BR-3/R cells. (DOCX 382 KB)

Additional file 2: Figure S2: The role of MTDH to trastuzumab therapy in breast cancer model. A and B. Representative images of tumors isolated from athymic nude mice bearing HER2 positive breast cancer; C. Tumor weight in different groups; D. Relative Ki67 expressions in different groups; E. Relative PTEN expressions in different groups. (DOCX 417 KB)

## Abbreviations

MTDH: Metadherin
HER2: Human epidermal growth factor receptor 2
PTEN: Phosphatase and tensin homologue deleted from chromosome 10
NF-κB: Nuclear factor kappa B
EMT: Epithelial-mesenchymal transition
Akt: Protein kinase B
PI3K: Phosphatidylinositol-3-Kinase
FOXO: Forkhead transcription factors of the O class.
