# Supplementary material for: MTDH mediates trastuzumab resistance in HER2 positive breast cancer by decreasing PTEN expression through an NFκB-dependent pathway
Source: BMC Cancer. 2014 Nov 24;14:869. doi: 10.1186/1471-2407-14-869 (PMC4254009; doi:10.1186/1471-2407-14-869)
Supplement: Supplementary file 1 — Additional file 1: Figure S1: Representative images of 5-ethynyl-2'-deoxyuridine (EdU) incorporation assay. MTDH was overexpressed in SK-BR-3 cells and knocked down in SK-BR-3/R cells. (DOCX 382 KB) [file 12885_2014_5053_MOESM1_ESM.docx]

Supplementary data


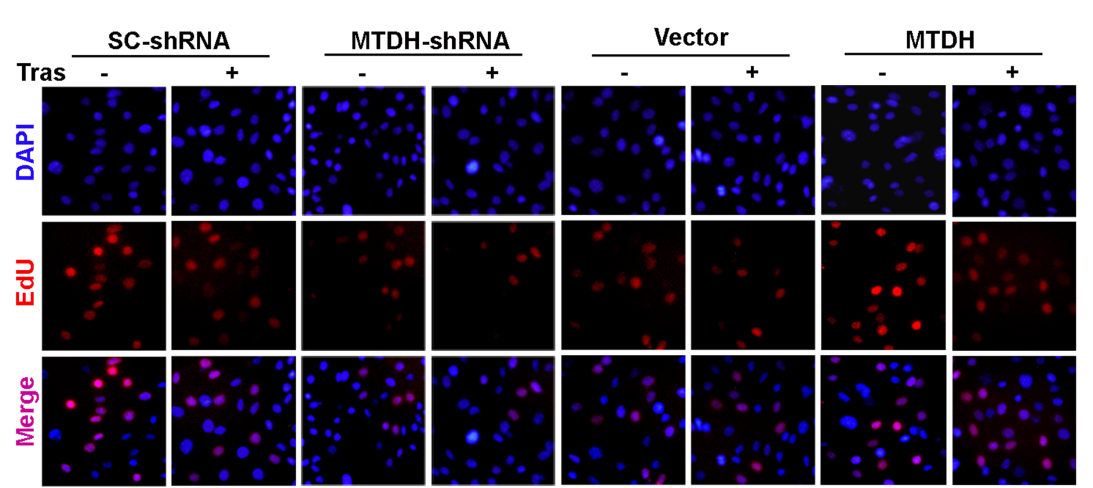


**Figure S1. Representative images of 5-ethynyl-2'-deoxyuridine (EdU) incorporation assay.** MTDH was overexpressed in SK-BR-3 cells and knocked down in SK-BR-3/R cells
