# Supplementary material for: MTDH mediates trastuzumab resistance in HER2 positive breast cancer by decreasing PTEN expression through an NFκB-dependent pathway
Source: BMC Cancer. 2014 Nov 24;14:869. doi: 10.1186/1471-2407-14-869 (PMC4254009; doi:10.1186/1471-2407-14-869)
Supplement: Supplementary file 2 — Additional file 2: Figure S2: The role of MTDH to trastuzumab therapy in breast cancer model. A and B. Representative images of tumors isolated from athymic nude mice bearing HER2 positive breast cancer; C. Tumor weight in different groups; D. Relative Ki67 expressions in different groups; E. Relative PTEN expressions in different groups. (DOCX 417 KB) [file 12885_2014_5053_MOESM2_ESM.docx]

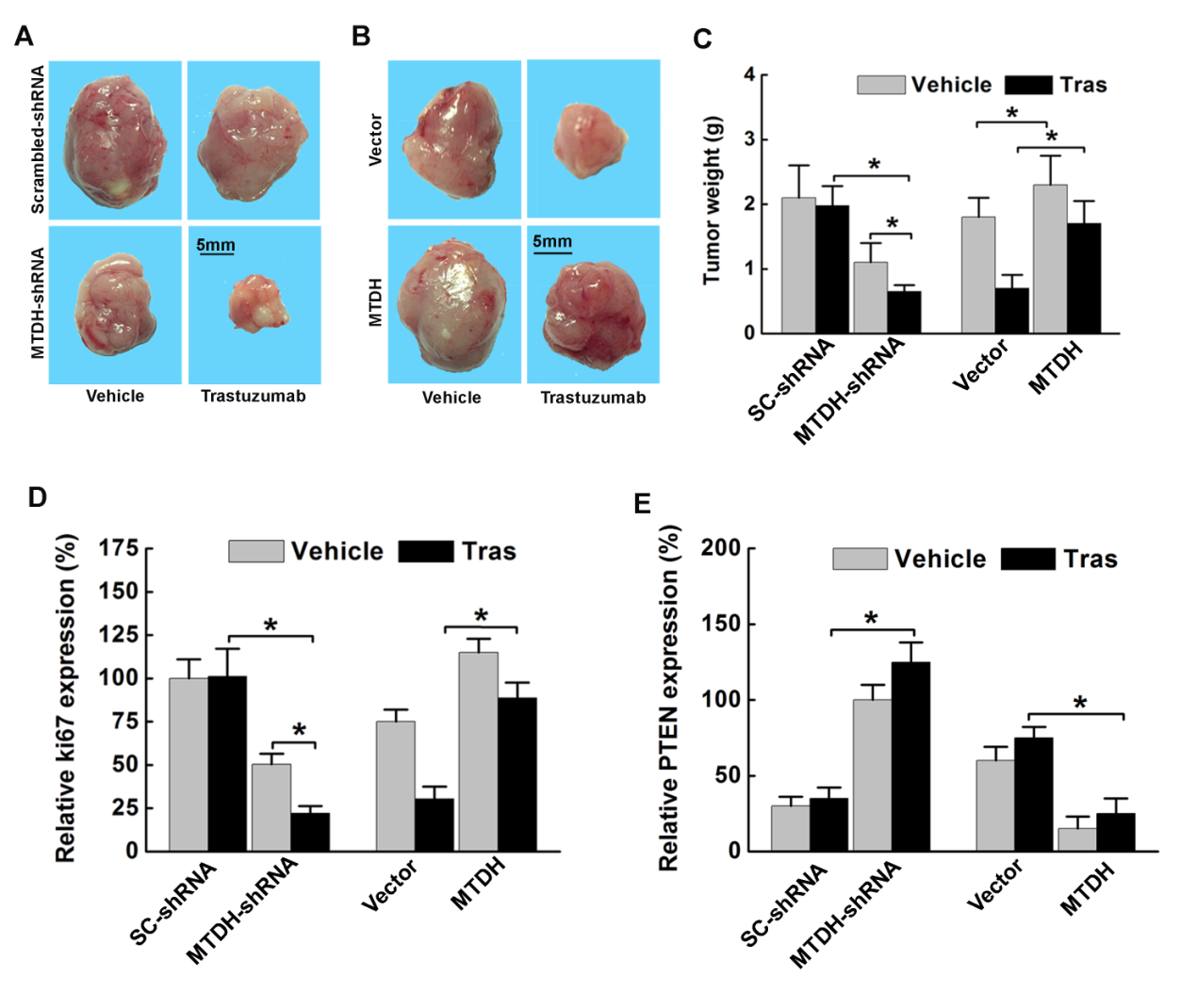


**Figure S2. The role of MTDH to trastuzumab therapy in breast cancer model.** A and B. Representative images of tumors isolated from athymic nude mice bearing HER2 positive breast cancer; C. Tumor weight in different groups; D. Relative Ki67 expressions in different groups; E. Relative PTEN expressions in different groups.
